# Role playing in human evolution: from life to art, and everything in between

**DOI:** 10.3389/fpsyg.2024.1459247

**Published:** 2025-01-06

**Authors:** Steven Brown

**Affiliations:** Department of Psychology, Neuroscience & Behaviour, McMaster University, Hamilton, ON, Canada

**Keywords:** role playing, acting, cultural evolution, emulation, conformity, pantomime

## Abstract

Role playing is a central, but underappreciated, process in human evolution. It is a feature not only of the theatrical arts, but of everyday social interactions. While some role playing is limited to enacting various personas of the self (e.g., wife, accountant, mother), others involve an impersonation of people. The most basic form of impersonation is proto-acting, which refers to a transient engagement in character portrayal, such as when we quote a friend during a conversation. During proto-acting, we “act as” some other person. However, there are other means of acting in a similar manner to another person in which we do not impersonate them, but merely *emulate* their behavior. This might happen when we learn a motor skill from a teacher or conform to the consumer choices of the masses. This follower-based process of “acting like” is a critically important mechanism in cultural evolution since it leads to social conformity and the homogenization of group behavior. I argue that the evolutionary transition from “acting like” (emulation) to “acting as” (impersonation) occurred via the emergence of pantomime and its narrative depiction of the actions of other people. This was probably the first step toward impersonating someone, leading initially to proto-acting and later to theatrical performance in human cultures. Overall, the study of human evolution needs to give greater consideration to role playing and its diverse manifestations in life and art.

## Introduction

In this article, I argue that role playing is an important, but underappreciated, phenomenon in the study of human evolution. A central aim of the article is to unite the study of role playing with that of cultural evolution. In particular, cultural evolutionary theorists propose that behaving in a similar manner to other people via conformity mechanisms is a strong force that supports the evolution of cooperation and cultural transmission. I argue that this capacity for acting *like* another person serves as a precursor for the process of acting *as* another person – in other words, impersonation – in both the theatrical arts and everyday life. I conclude with the proposal that impersonation has its roots in the evolution of the human capacity for pantomime during narrative communication.

## The role playing spectrum

**Figure 1:**
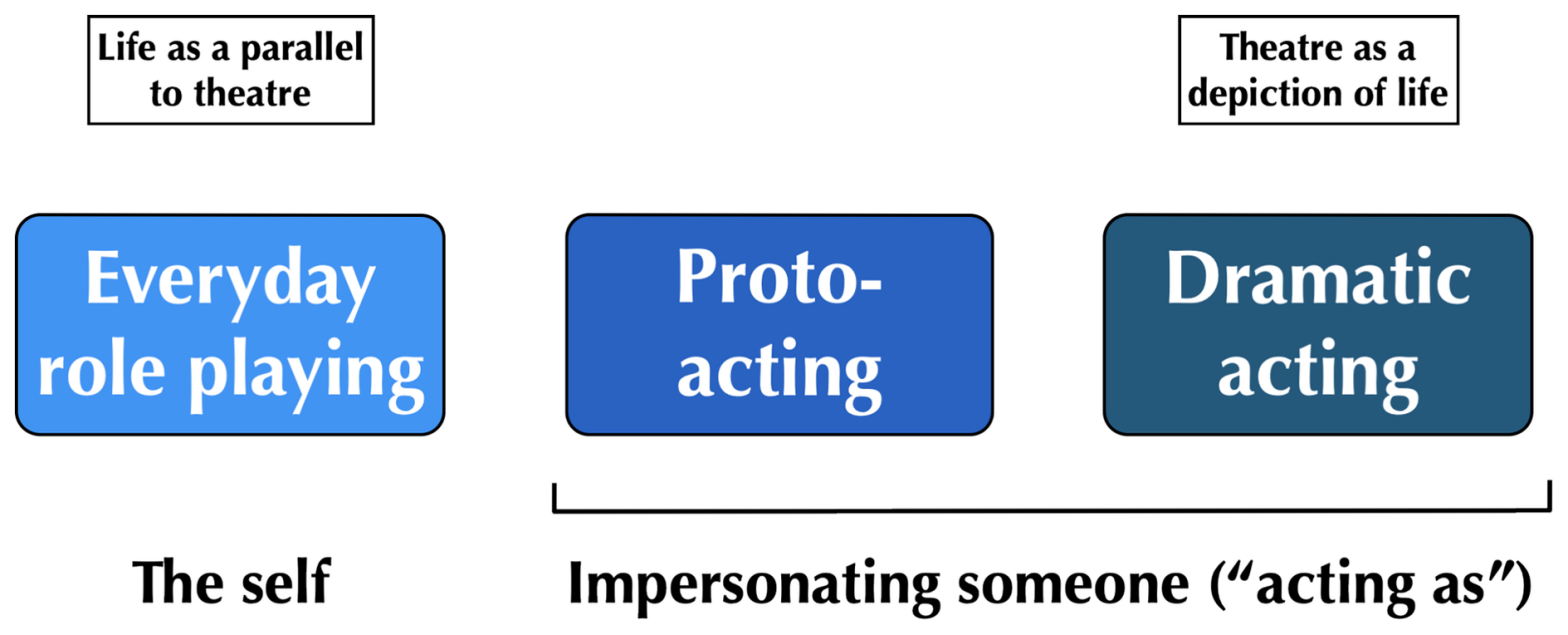
The role playing spectrum. The figure outlines the categories of role playing in life, art, and diverse phenomena that sit in between them in which people transiently impersonate other individuals. This intermediate category maps onto the concept of proto-acting from Brown (2017), involving various forms of personal mimicry in both life and art. Note that there is a categorical distinction between everyday role playing and the other two categories in that the latter involve an impersonation of other people, not merely an enactment of personas of the self. This is referred to in the text as “acting as” some other person.

For more than 2,500 years, role playing has been associated with the portrayal of fictional characters by trained actors in the theatrical arts (Aristotle, 335BC/1996). However, theoretical developments in the social psychology of the mid-20th century, spearheaded by thinkers like Erving Goffman, led to the emergence of the concept of role playing in everyday life, known as the dramaturgical perspective (Goffman, 1959; Appelrouth and Edles, 2011; Shulman, 2017). This perspective argues that there are strong parallels between everyday life and theater (see Figure 1). As Goffman (1959) writes, “all the world is not, of course, a stage, but the crucial ways in which it is not are not easy to specify” (p. 72). A big part of the role playing of everyday life is what Goffman refers to as “impression management” or the strategies that people employ to convince others that they are who they want them to believe they are. This involves the use of the same devices of stagecraft that an actor would bring to the portrayal of a character, including “insignia of office or rank; clothing; sex, age and racial characteristics; size and looks; posture; speech patterns; facial expressions; body gestures; and the like” (p. 24).

The roles that people play in everyday settings are not fictional characters, but instead “personas” associated with people’s functional roles in different social contexts (Shulman, 2017). For example, someone who performs the role of doctor in a hospital setting transitions to the role of customer when they enter a car-repair shop, a change from a leader role to a follower role. Personas vary in their level of dominance and their degree of cooperativeness, just as with fictional characters (Berry and Brown, 2017). An individual’s full set of personas comprise what Neisser (1988) refers to as the “conceptual self.” Goffman’s examples of dramaturgy focus on a number of common professions in contemporary society, such as doctor and hotel worker. While most role playing in human life is genuine, people occasionally engage in what Goffman refers to as “misrepresentation” when they attempt to deceive others by exaggerating or falsifying aspects of their presented persona.

The remaining two categories in the role playing spectrum involve a categorical shift from portraying oneself toward engaging in an act of *impersonation* in which a person presents themselves as someone whom they are not. In other words, they are “acting as” someone else. This assumes two forms, what I will refer to as proto-acting and dramatic acting. Looking to the right end of the spectrum in Figure 1, we find forms of dramatic acting in which professional actors make strong commitments to portraying individual characters in large-scale dramatic works, such as in stage plays and feature films. Such actors present themselves as characters whom they are not during extended performances, and interact with other people on stage as these characters (Blair, 2008; Lutterbie, 2011; Kemp, 2012; Mirodan, 2019; Krumholz, 2023). In general, theater is a depiction of everyday life, conveyed by fictional characters engaged in conversations and actions, while enacting plots containing social conflicts. Goffman (1959) refers to the behavior of actors as forms of “licensed” misrepresentation since their portrayal of fictional people is known to audiences and is not an attempt to deceive them. The craftsmanship that actors bring to misrepresenting themselves through character portrayal is something that is aesthetically appreciated by theater audiences.

From the standpoint of the argument being made in this article, it is not important how an actor is able to get in character or the cultural contexts in which acting occurs. It does not matter if the portrayal is achieved through pure mimicry or the most creative workings of the imagination. And it should not matter whether the actor truly “becomes” the character by mentalistically transforming into them, or if they instead employ a gestural approach that produces a surface representation of the character in the absence of psychological engagement. The main psychological point is that there is a categorical distinction between self and other, and that actors present themselves in public as people whom they are not for extended periods of time. These are not personas of the self, but instead completely other people. It is interesting to note that many viewers of films and television series enter into parasocial relationships with the depicted characters (Shackleford, 2020), and that they can even come to conflate the actor with the character whom they are portraying (Goldstein and Filipe, 2018). The latter can happen to actors themselves in situations of “post-dramatic stress syndrome” (Seton, 2006), especially in cases where a film actor has chosen to live in character during the several months of a film shoot.

Dramatic acting can occur comparably in dance dramas, such as in the ballet *Romeo and Juliet*. In such dramas, the dancers function as true actors by portraying fictional characters, except that they do not typically speak (Brown, 2022b, 2024). The conventions of classical dance are such that the actors are mute, conveying the characters’ actions and emotions using movement and gesturing alone. Aside from this limitation, the dance-based actor is just like a typical dramatic actor in that they wear costumes, use props, and move about a stage containing sets. When Romeo seeks out Juliet in the balcony scene of the ballet, Juliet is situated on a balcony, and when Romeo duels with Tybalt during the duel scene, the dancers use swords.

Dramatic acting is not the only form of impersonation that occurs in human life. It merely represents one extreme in which the performers are trained actors engaged in ritualized public performances to an audience, often times speaking lines written by professional playwrights. Sitting in between the role playing of everyday life and that of the theatrical arts is an intermediate category along the spectrum (see Figure 1). In Brown (2017), I used the term “proto-acting” to refer to *transient acts of character portrayal*. The defining feature of proto-acting is impersonation (i.e., personal mimicry), but this occurs on a much shorter time-scale than in dramatic acting, and it often manifests itself in everyday contexts, rather than in stage performances to a mass audience. These are situations in which a person changes their voice, gestures, facial expressions, and/or linguistic content to convey the fact that they are speaking or moving as a person whom they themselves are not.

The forms of proto-acting are quite diverse, extending across life and art. Perhaps the most fundamental form in everyday life is quoting someone during a conversation (Blackwell et al., 2015; Clark, 2016; Stec et al., 2016). During quotation, we temporarily *become* some other person—through changes in our voice, words, facial expression, posture, gesturing, and/or manner of moving—and then revert back to our selves after the quotation is done. Something similar happens when a parent reads a bedtime story to their child and portrays the characters during the segments of dialog (Matharu et al., 2021). For example, they may alter the pitch of their voice in different ways to sound like Little Red Riding Hood and the Wolf during their initial encounter in the story, and then again later when the Wolf proto-acts as Riding Hood’s grandmother in order to deceive her. An important developmental form of proto-acting is pretend play in children (Walton, 1999; Harris, 2000; Lillard et al., 2011), unquestionably the precursor of theater in the world of adults. Therapeutic forms are found in psychodrama and drama therapy, where the client proto-acts as either some familiar person or a fictional character (Kipper and Ritchie, 2003; Orkibi and Feniger-Schaal, 2019; Sajnani, 2021). A rapidly growing form of proto-acting in everyday life is people’s engagement in role-playing video games and board games (Ryan, 2015; Tu et al., 2023). Other forms of proto-acting involve stage performances, such as impressionism, ventriloquism, puppetry, and sketch acting, where the performers engage in short-term portrayals of characters, whether these be of real or fictional people.

## Acting like vs. acting as

**Figure 2:**
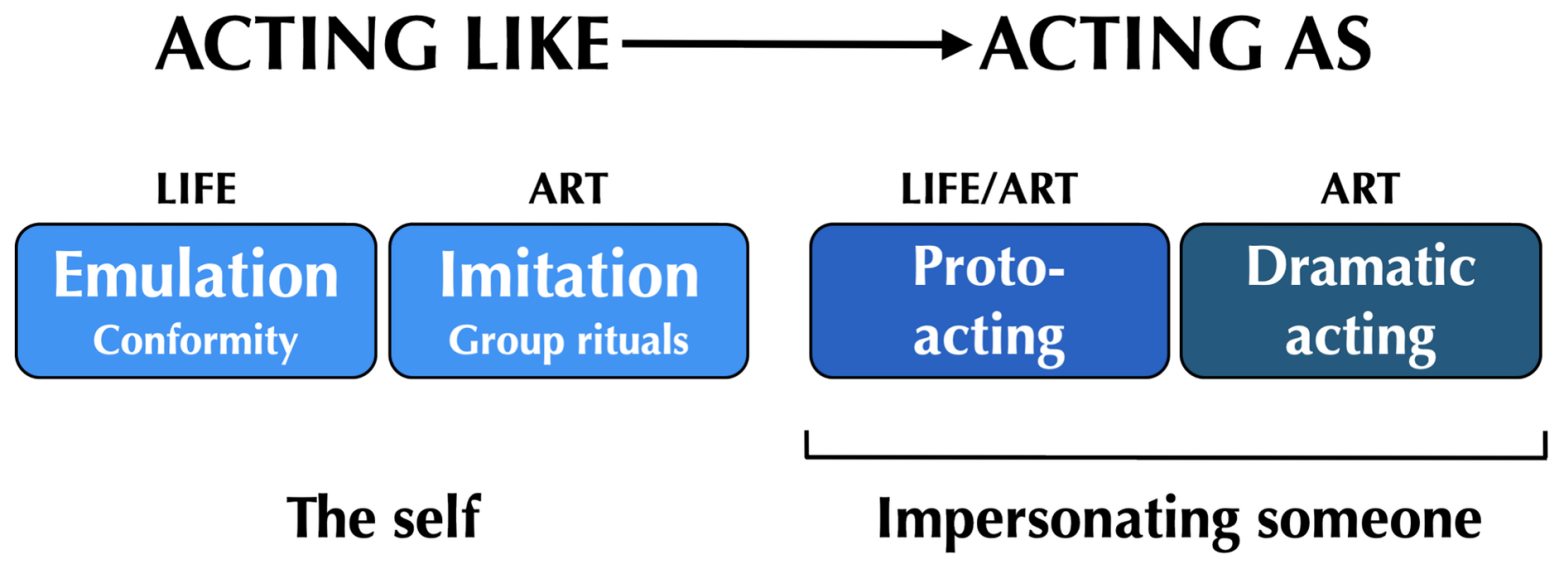
Acting like vs. acting as. “Acting like” is proposed as a phenomenon of role playing in both life and art. The “life” manifestation relates to people’s general tendency in everyday interactions to emulate the behavior of those around them through conformity mechanisms in order to support cooperation and social harmony. The “art” manifestation is seen in the unison performance arrangement of music and dance in group-coordination rituals. “Acting as” (right side) covers the topics mentioned earlier about proto-acting and dramatic acting (see Figure 1). Here, a person engages in an act of impersonation in order to present themselves in a public setting as someone whom they themselves are not. This can occur in both life and art. The color coding of the boxes is the same as in Figure 1.

These phenomena of personal mimicry—whether through short-term proto-acting or full-fledged dramatic acting—constitute what I will refer to as “acting as,” which are times when people present themselves in public as someone whom they are not. As mentioned above, this can be as transient as quoting a friend during a conversation and as extensive as performing the role of Romeo or Juliet during a 3-h stage performance or a 3-month film production. However, I would like to introduce a new distinction, one that applies to the role playing of everyday life, but in ways that are very different from what Goffman described in his analysis of common professions. I will call this process “acting like” to distinguish it from “acting as,” as shown in Figure 2.

My major aim of introducing the “acting like” concept is to unite the study of role playing with that of cultural evolution, and more specifically with the importance of social emulation—in other words, *acting like other people*—for the evolution of culture. Humans have achieved a highly sophisticated form of culture compared to all other social animals (Boyd and Richerson, 2005; Mesoudi, 2011; Richerson et al., 2016). This is characterized not only by a complexification and diversification of material culture (e.g., tools, infrastructure), but by a comparable complexification of social organization, leading to the large-scale societies of modern times (Turchin, 2013). The human capacity for culture involves not only the ability to maintain traditions across generations (Jagiello et al., 2022), but also the talent for generating novelty although acts of creativity (Csikszentmihalyi, 1988; Fogarty et al., 2015; Carr et al., 2016), as seen in the striking acceleration in product innovation across all domains of technology in modern times (Wilf, 2015).

Cultural evolutionists tell us that the most important mechanism that enables culture is *social learning*, which is the ability to faithfully transmit information and/or objects from person to person across generations via processes such as imitation, emulation, and teaching (Boyd and Richerson, 1985, 2005), where emulation is distinguished from imitation in that, while imitation is about copying the *process* of performing an action, emulation is about copying the *end-product* of that action (Tomasello et al., 1993; Heyes, 2021). Social learning from other people is contrasted with individual-level learning, such as trial-and-error learning, which is thought to be far less efficient at maintaining and transmitting information across people (Boyd and Richerson, 1985; Mesoudi, 2011). Because of social learning, people do not have to “reinvent the wheel” each generation, but can instead inherit technical knowledge from their predecessors about how to construct wheels. This allows wheels to be transmitted faithfully across generations, and can also lead to cumulative improvements over time (Tomasello et al., 1993; Tennie et al., 2009) such that the stone wheel of 4,000 BCE becomes the vulcanized rubber tire of modern vehicles. A key outcome of social learning is that it has enabled humans to inhabit all of the extreme environments of the earth (Boyd et al., 2011). People’s ability to thrive in such environments is not due to genetic differences across populations, but instead to the cultural transmission across generations of survival strategies for flourishing in these habitats, such as knowledge related to finding food sources, evading predators, constructing tools, and finding shelter.

Beyond this material aspect of cultural evolution is the critical importance of cooperation for the maintenance of social organization. Most of the key achievements of culture require not just social learning but collaborative actions that are carried out by groups of people. One of the biggest drivers of social cooperation in cultural evolution is inter-group competition, which is a selection pressure that enhances mechanisms that lead people to cooperate with one another and make sacrifices on behalf of their social group. Cultural evolutionary theory argues that such mechanisms operate best when people strive to be most similar to one another through *conformity* mechanisms, thereby increasing their self-identification with the group (Henrich and Boyd, 1998; Mesoudi and Lycett, 2009). Conformity essentially homogenizes human behavior. This is thought to be adaptive for cultural evolution because it dampens within-group differences between people, thereby amplifying between-group differences, which themselves contribute to the evolution of cooperation through cultural, and potentially genetic, means (Boyd and Richerson, 2005; Richerson et al., 2016; Sober and Wilson, 1998). Conformity is a process of “acting like” others around us. It is defined as copying the most prevalent behavior in a population (Henrich and Boyd, 1998; Mesoudi and Lycett, 2009). In conformity, we emulate other people, from their manners of behaving to the products that they consume (e.g., clothing, music, cars). “Acting like” in this sense is a form of social emulation whereby we model our actions on the behaviors of those around us.

“Acting like” has broad implications for all forms of *following behavior* in human cultures.^1^ Key manifestations of this include: (1) conformity in consumer behavior, leading to winner-take-all distributions in which a tiny proportion of variants dominate a given domain (Frank and Cook, 1996; Acerbi, 2016); (2) the cooperation and coordination of joint instrumental actions in which people work together to achieve common goals (Knoblich and Sebanz, 2008; Keller et al., 2014), such as in building infrastructure or waging war (Turchin, 2013); (3) norm psychology, in which people intentionally act like other members in their community in order to abide by common standards of behavior (Mathew et al., 2013); (4) mechanisms of group affiliation in which people come to feel connected with those in their social group by acting in manners similar to them (Haun and Over, 2013; Launay et al., 2016; Savage et al., 2021); and (5) the “receiver” role during conversation, in which the receiver follows the narrative thread of the sender and fashions a reply that is concordant with it (Levinson, 2016). These are among many other examples of follower behaviors in everyday role playing in which people act like others in order to achieve a sense of group cohesion, cooperativeness, and social harmony. This is manifested psychologically in the phenomenon of “homophily,” in which people come to develop a social preference for interacting with and emulating others whom they perceive as being similar to themselves (Haun and Over, 2013).

There is a second major context in which “acting like” occurs in human life, and it is in domain of the arts, in particular groupwide coordination rituals. This applies to practices of group chorusing and dancing that are done *in unison* such that every person performs the same part at the same time, for example when people sing “Happy Birthday” at a party or dance a bunny hop at a wedding. Donald (1991) refers to coordinated rituals of this kind in which people match their behavior to one another as forms of “group mimesis,” highlighting the inherently imitative nature of these behaviors. Such group behaviors are a ritualization of “acting like.” In fact, Donald proposes a stage in hominin evolution that he calls Mimetic Culture that is characterized by a complex suite of behaviors involving “acting like” (Donald, 1991, 2013). It should be pointed out that not all performance arrangements of music and dance are unison arrangements. However, unison is probably the most human-specific format of these activities (Brown, 2022a, 2022b).

The proposal that coordination rituals performed in unison constitute a form of “acting like” provides a novel perspective on the important concept of “entrainment” in the study of human social interaction, where entrainment connotes a synchronization of action between two or more people (Sebanz et al., 2006; Knoblich and Sebanz, 2008; Schmidt and Richardson, 2008; Keller et al., 2014). While entrainment focuses on the synchronous nature of such interactions in time, “acting like” emphasizes the spatial uniformity of the melodic lines (group chorusing) or choreographic patterns (group dancing) across the performers of these rituals. Radcliffe-Brown (1922) argued that unison chorusing represents a group of people speaking as if with one voice, and that unison dancing represents a group of people moving as if with one body. These are the most “organismal” forms of group behavior in the repertoire of human social interaction. As mentioned above, such homogenization of group behavior is evolutionarily adaptive since it dampens within-group differences between people, thereby enhancing salient cultural differences between groups. To summarize, “acting like” is a follower-based form of role playing in which people imitatively behave in a similar manner to others around them—whether spontaneously or in a directed manner—often times as a means of fitting into a group and/or enhancing the integrity of a group. This is different from impersonation. In “acting like,” people emulate others without portraying them.

## The evolution of acting

**Figure 3:**
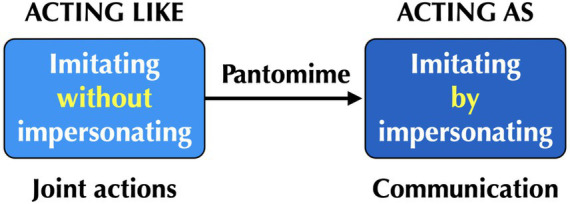
The evolution of dramatic acting. Evolution of the capacity for pantomime is proposed here as the catalyst for the transition from imitating people without impersonating them (“acting like”) to imitating people by impersonating them (“acting as”), where the latter leads to the evolution of proto-acting and ultimately dramatic acting.

I would like to consider the implications of these ideas for the evolution of acting as a newly-evolved human behavior. I argued in a previous article that dramatic acting evolved as a ritualized and performative offshoot of proto-acting (Brown, 2017). In addition, I argued that proto-acting itself evolved as a derivative of *pantomime*^2^—in other words, iconic gesturing that structurally resembles the action or object being depicted (Arbib, 2012; Żywiczyński et al., 2018; Brown et al., 2019b; Zlatev et al., 2020)—as a novel human capacity to mimic other people, permitting both gestural and vocal imitation of people during acts of communication. To understand this emergence, we need to consider the behavioral transition from (1) imitating people *without* the goal of impersonating them—such as during joint actions like group chorusing and dancing—to (2) the goal of imitating them by “becoming” them as characters (Figure 3). This is basically a transition from “acting like” to “acting as.” Regarding the former, there are ample examples of animals that engage in joint actions in which they behave imitatively in order to stay together. Consider the movement of a flock of birds, a school of fish, or a procession of migrating wildebeest. These animals move in a similar manner to one another so as to maintain the integrity of their group. This is similar to the cases of chorusing and dancing in group-mimetic rituals in humans.

However, the animals in these situations are not “acting as” the other members of their group; they are not trying to impersonate them, but are simply engaging in a matched action to conform with them. There thus had to have been a transition from “acting like” to “acting as” in human evolution. I continue to contend that the best means for having achieved this behavioral change was through the evolution of the capacity for pantomime during acts of interpersonal communication (Figure 3). This is especially true for the full-body form of pantomime in which one depicts the actions of other people in egocentric space (Arbib, 2012; Żywiczyński et al., 2018; Brown et al., 2019b; Zlatev et al., 2020), such as when we mime a tennis serve. Pantomimic depictions tend to be holistic, multimodal, improvised, open-ended, and communicatively complex (Żywiczyński et al., 2018). The capacity to create gestural and vocal depictions of other people during mimetic acts of narrative communication was probably the initial evolutionary step toward “acting as” someone, leading first to proto-acting and ultimately to dramatic acting in human evolution, as shown in the role playing spectrum in Figure 1. I have argued elsewhere that pantomime served as a reasonable precursor for the narrative arts as a whole, including the visual arts and the theatrical arts, the latter including narrative forms of dance (Brown, 2024).

## Toward an empirical approach to role playing

The present article has presented a theoretical approach to the origins of role playing in humans, one that has attempted to incorporate theorizing in cultural evolutionary studies. However, the model presented here interfaces with behavioral and psychological findings in numerous domains that can be used to validate the ideas presented here.

### Everyday role playing

In animals, everyday role playing can be explored by examining topics such as dominance hierarchies (Tibbetts et al., 2022) and the division of labor in workgroups (Page and Erber, 2002; Cooper and West, 2018), including leader/follower dynamics. For humans, in addition to such topics, it is important to develop a better understanding of the dramaturgical devices that people employ during everyday role playing. This could come from social psychological analyzes of behavioral interactions during social role playing or from psychological and physiological approaches to how people change their mindset and manners of acting when playing different social roles. This includes applied dramaturgical approaches to, for example, leadership training in business (Boje et al., 2015; Tawadros, 2015).

### Proto-acting

Proto-acting touches on a diversity of phenomena involving impersonation, most of which have not been empirically studied by psychologists. This includes ventriloquism and impressionism, where performers move quickly in and out or character in a seamless manner. There is experimental work on how storytellers of fairy tales modify their vocal pitch during the impersonation of the multiple characters in a story (Doukhan et al., 2011; Matharu et al., 2021), but such studies are quite rare. There needs to be more work on how individuals transiently fall in and out of character, such as when they quote someone during a conversation (Stec et al., 2016), which might be the most common form of proto-acting in everyday life. Perhaps the largest emerging phenomenon of proto-acting is found in role-playing video games (Tychsen et al., 2006; Hitchens and Drachen, 2006; Leménager et al., 2014; Shulman, 2017), in which players demonstrate the psychological features of acting (Tu et al., 2023). Finally, there is a significant developmental literature on pretend play in children (Walton, 1999; Harris, 2000; Lillard et al., 2011). At a more general level, play is a widespread behavior across animal species (Graham and Burghardt, 2010; Toomey, 2024), although pretend play might be a form that is specific to humans.

Can we identify phylogenetic precursors of acting in non-human animals? On the one hand, there is a voluminous literature devoted to the topic of *deception* in animals (Sarkadi et al., 2021) that may provide insight into the roots of proto-acting and mentalizing. This includes interesting phenomena such as Batesian mimicry (Joron and Mallet, 1998), whereby, for example, a non-poisonous but palatable species develops the appearance of a related poisonous species that announces its harmfulness through defensive coloration (the latter being called aposematism). Next, Russon and Andrews (2011) described 18 instances of spontaneous pantomime in forest-living rehabilitant orangutans living in Indonesia, 14 addressed to humans and four to other orangutans. These often conveyed an intent for another individual to perform the action modeled by the pantomime. This resembles what is perhaps the only study that has suggested the occurrence of impersonation in a non-human animal. Matsuzawa (2020) claimed that a wild chimpanzee in Guinea engaged in a portrayal of local human villagers by putting on a grass head cushion (used for transporting objects on one’s head) and walking in the bipedal manner of a human. Much work is needed to explore if the roots of acting can be found in pervasive animal behaviors involving deception, mimicry, and perhaps symbolic pantomime.

### Dramatic acting

The central research question for work on dramatic acting is about the methods that actors use to get into character, and whether these methods are more psychological or gestural in orientation, or a combination of the two (Stanislavki, 1936; Kemp, 2012). While most of this work is carried out in the non-experimental context of actor training, there is an emerging field devoted to the biological basis of acting, including studies of expressive modalities (Berry and Brown, 2019, 2022; Berry et al., 2022), brain activations (Brown et al., 2019a; Greaves et al., 2022; Lim et al., 2024a, 2024b), and hormonal changes (Berceanu et al., 2024). This complements work on mainstream topics in cognitive neuroscience such as imitation (Caspers et al., 2010; Papitto et al., 2020), pantomime (Molenberghs et al., 2010; Osiurak et al., 2021), action observation (Caspers et al., 2010; Papitto et al., 2020), theory-of-mind (Denny et al., 2012; Schurz et al., 2014), empathy (Decety and Holvoet, 2021), and self-processing (Heatherton, 2011) that are extensively studied outside of the realm of acting and role playing. Finally, looking beyond adults, developmental studies examine the impact of acting training on childhood development (Goldstein et al., 2013; Goldstein, 2024).

## Why do humans act?

Why have humans evolved the unique capacity to impersonate others? In what way can this be thought of as being an adaptive behavior from a Darwinian perspective? A competition-based view of impersonation might focus on acts of deception and defensive mimicry as general, cross-species means for misrepresenting the self, both to conspecifics and predators, much the way that we think about the actions of imposters in human societies who attempt to deceive and manipulate people. In fact, a major topic of study in human evolutionary psychology is that of “cheaters” (Cosmides and Tooby, 1992), in other words individuals who falsely present themselves as cooperators, even though they do not actually contribute resources to collective efforts, but instead parasitize the group.

However, when it comes to human acting, we typically consider impersonation to be a *cooperative* action—what Goffman calls “licensed misrepresentation”—rather than an attempt to manipulate people. I argued that the drive for conformity might have served as an initial form of imitation and emulation, and that this supported the evolution of cooperation by homogenizing group behavior and accentuating salient differences between groups. I called this “acting like,” and this includes mimetic group-level behaviors like unison chorusing and dancing. However, there is no impersonation here, just an imitative convergence of behavior across group members. In order to depart from the self, people could certainly engage in mechanisms of deception, but there would seem to be more-cooperative routes to the achievement of impersonation during social communication. These include the capacities for imitation (to learn motor skills), emulation (to model oneself on successful individuals), pantomime (to communicate narrative information), and social play (to practice risky behaviors in a safe manner). I argued that the most fundamental form of impersonation is proto-acting, which is a short-term departure from the self, but one that is licensed by receivers, rather than an attempt to deceive them. This serves primarily a *communicative* function in human interactions, conveying narrative information about a person or event, as occurs commonly during quotation in conversation. Hence, the primordial function of acting might have been the same one that predominates today: to convey salient social information about people and events involving them, although a competitive function in deception needs to be acknowledged as well.

Using proto-acting as a foundation, human cultures came to develop dramatic acting as a more performative version of impersonation once theatrical rituals evolved, initially as part of religious ceremonies (Turner, 1969; Schechner, 1974, 2013), where acting might have been used to depict deities and mythic characters. We know from the study of ancient Greek theater that plays were originally performed by a single actor. Aeschylus is credited with introducing a second actor into the performance of plays in the 5th century BCE (Storm, 2016), something that was considered as a major innovation at the time. In ancient Rome, the pantomime was someone who performed all (= panto) of the characters in a story through elaborate forms of dancing and gesturing, “transforming himself from one role to another assisted by little more than a change of mask” (Hall, 2013, p. 451). Theatrical rituals are present in most, if not all, world cultures (Schechner, 2013). These performances include the impersonation of not only of people but animals as well, and they incorporate dance in addition to—or in place of—spoken language (Sachs, 1937; Knauft, 1985). Finally, religious rituals in many world cultures involve the practice of possession trance (Rouget, 1985), and this might be conceptualized as a form of proto-acting. In fact, the process of acting might be similar to possession in that both can may be underlain by a “loss of self” (Brown et al., 2019a).

## Conclusion

The goal of this article was not only to revisit the role playing spectrum of everyday role playing, proto-acting, and dramatic acting (Brown, 2017), but to introduce the new concept of “acting like” as a means of uniting the study of role playing with the voluminous literature devoted to cultural evolution. In particular, conformity has achieved a status of central importance in theories of cultural evolution, and this can be reasonably seen as a form of everyday role playing in which the focus is on emulating the behavior of others. I also proposed that group-coordination rituals related to chorusing and group dancing demonstrate the process of “acting like” when they are performed imitatively in a unison manner, leading to the most organismal forms of human behavior. The social role of “follower” is one of the most significant forms of everyday role playing for the evolution of human culture. This is so because it homogenizes group behavior, thereby accentuating inter-group differences. Finally, I argued that the transition from “acting like” (emulation) to “acting as” (impersonation) was catalyzed by the evolution of the capacity for pantomime and its iconic depiction of the whole-body actions (and potentially vocalizations) of other people during mimetic acts of communication. This led to proto-acting as a form of narrative communication and ultimately theatrical performance in human evolution. Overall, role playing is a central, but underappreciated, process in human evolution that merits further investigation in studies of the evolution of culture.

## Data Availability

The original contributions presented in the study are included in the article/supplementary material, further inquiries can be directed to the corresponding author.

## Footnotes

^1^Leaders, in contrast to followers, do not typically act like others, but instead forge their own manner of behaving, often serving as a role model for others. So, “acting like” is more strongly associated with following than leading, even though leading is a form of everyday role playing.

^2^Note that an evolutionary discussion of pantomime as iconic gesturing during communication should not be confused with the artform of mime theatre, which employs pantomimic gesturing in stage performances. While mime actors are mute by convention, there is no reason to believe that the emergence of pantomime during human evolution had to have excluded vocalization (Donald, 1991; Zlatev et al., 2017, 2020; Brown, 2024).
